# Resonance Properties in Auditory Brainstem Neurons

**DOI:** 10.3389/fncel.2018.00008

**Published:** 2018-01-24

**Authors:** Linda Fischer, Christian Leibold, Felix Felmy

**Affiliations:** ^1^Zoologisches Institut, Stiftung Tierärztliche Hochschule Hannover, Hannover, Germany; ^2^Department Biologie II, Ludwig-Maximilians-Universität München, Munich, Germany; ^3^Bernstein Center for Computational Neuroscience Munich, Munich, Germany

**Keywords:** membrane resonance, auditory brainstem, MSO, LSO, VNLL

## Abstract

Auditory signals carry relevant information on a large range of time scales from below milliseconds to several seconds. Different stages in the auditory brainstem are specialized to extract information in specific frequency domains. One biophysical mechanism to facilitate frequency specific processing are membrane potential resonances. Here, we provide data from three different brainstem nuclei that all exhibit high-frequency subthreshold membrane resonances that are all most likely based on low-threshold potassium currents. Fitting a linear model, we argue that, as long as neurons possess active subthreshold channels, the main determinant for their resonance behavior is the steady state membrane time constant. Tuning this leak conductance can shift membrane resonance frequencies over more than a magnitude and therefore provide a flexible mechanism to tune frequency-specific auditory processing.

## Introduction

The membrane potential of nerve cells exhibits rich intrinsic dynamics to facilitate the function of the embedding neural circuit (Fransen et al., 2004; Nelson et al., 2005; Wu et al., 2005). Resonance properties are a specific well-studied example for such an intrinsic dynamical feature (Hutcheon and Yarom, 2000) and have been hypothesized to underly rhythmogenesis (Leung and Yu, 1998) and efficient sensory processing (Chacron et al., 2001; Remme et al., 2014). Biophysically, membrane potential resonances are generally assumed to result from active subthreshold conductances, such as hyperpolarization-activated cyclic nucleotide-gated cation channels (HCN) (Boehlen et al., 2013; Hu et al., 2016) or low-threshold potassium channels (Beraneck et al., 2007; Hsiao et al., 2009). Thereby, subthreshold voltage deflections gate the opening of one or several of these channels that then mediate a transmembrane current forcing the voltage back to rest with a given time constant. Traditionally it is thought that these subthreshold channel kinetics implement a high pass filter, since they would remove constant components of the input current (Hutcheon and Yarom, 2000). The resonance property would then result from a combination of this active high-pass with the low-pass properties of the passive membrane resulting from a finite input resistance.

In this paper, however, following Richardson et al. (2003), we argue that the two ingredients, active high pass and passive low-pass are not independent properties, but rather arise both from the steady state of the membrane, which particularly determines the membrane time constant. This is because, at rest, both open passive and open active channels contribute to the input resistance and thus low-pass and high-pass properties are interrelated. To show that this is a general principle of subthreshold resonance in the auditory brainstem, we have analyzed resonance properties of five different populations of neurons, cells from gerbil medial superior olive (MSO) of two different age groups (P15: shortly after hearing onset, >P60: fully matured), lateral superior olive (LSO) neurons from mouse and rat, and neurons from gerbil ventral nucleus of the lateral lemniscus (VNLL) (all mature). Thus, our sample consists of neurons carrying out different auditory computations in the low- and high frequency domain. Although sampled from three different species, two different age groups, and three different nuclei, the resonance frequencies of the neurons can all be explained by the same simple dynamical model that only depends on one fit parameter to distinguish between the groups of cells. Most importantly, the observed large variability of resonance frequencies is indeed largely accounted for by the steady state membrane time constant (see also Schneider et al., 2011), i.e., the density of subthreshold channels that are open at rest. This finding predicts that neuronal resonance properties are present in all neurons where active subthreshold conductances yield leaky membranes.

## Methods

### Animals and preparation

Recordings were made in postnatal day (P) 19–25 (average: 21.5) B57BL6/6N mice, Mongolian gerbils (VNLL: P24–27, average: P25.5; MSO: P15/16 average 15.5 and >P60) and Brown Norway rats (P18–P27 average: 23.4) of either sex. Mice were purchased from Charles River or taken from our own breeding colonies; gerbils were obtained from own breeding colonies and rats were ordered from Charles River (Sulzfeld, Germany).

All experiments complied with institutional guidelines, and national and regional laws.

Animals were decapitated in deep isoflurane anesthesia. Brains were quickly removed in preparation solution containing (in mM) D-Saccharose 120, NaCl 25, NaHCO3 25, NaH2PO4 1.25, KCl 2.5, D-Glucose 25, L-Ascorbic acid 0.4, Myo-Inositol 3, Na-pyruvate 2, MgCl2 3, CaCl2 0.1 at a pH 7.4 and was oxygenated with 95% O2 and 5% CO2. After brains were trimmed 120 μm (MSO) or 180–200 μm (LSO and VNLL) thick sections were cut using a vibratome (Leica VT1200, Leica Microsystems GmbH, Germany). Slices were incubated for about 45 min at 34–35°C in extracellular recording solution containing (in mM) NaCl 125, NaHCO3 25, NaH2PO4 1.25, KCL 2.5, D-Glucose 25, L-Ascorbic acid 0.4, Myo-Inositol 3, Na-pyruvate 2, MgCl2 1, CaCl2 2 oxygenated with 95% O2 and 5% CO2.

### Data acquisition

Slices were transferred into the recording chamber integrated into upright BX50 or 51 WI Olympus microscopes and continuously perfused with recording solution. Data were acquired at near physiological temperatures of 34-36°C. Electrophysiological recordings were carried out with an EPC 10/2 amplifier (HEKA, Lambrecht/Pfalz, Germany). Electrode resistances were constrained between 3 and 5.5 MΩs. Stimulus generation and presentation was controlled by the PatchMaster software. Visual identification of brain structures and individual cells was carried out by CCD-cameras (TILL-Imago VGA, Retiga 2000DC) controlled by TILLvisION imaging system (FEI Munich GmbH, Munich, Germany). Constant background conductances were injected with an analog conductance amplifier (SM-1 Cambridge Conductance, Royston UK). Throughout these conductance clamp experiments, reversal potential of the leak was set to resting potential of a given cell. Recordings were performed in whole-cell configuration using an intracellular solution containing (in mM) K-gluconate 145, KCl 4.5, HEPES 15, Mg-ATP 2, K-ATP 2, Na2-GTP 0.3, Na2-phosphocreatine 7, K-EGTA 0.5, Alexa 488/594 0.05. Alexa labeling was used to control for cell localization within appropriate brain structure. Data were acquired with 20 kHz for ZAP stimulation (see ZAP Stimuli) and hyperpolarizing current steps. Only for neurons of >P60 MSO the sample frequency for hyperpolarizing steps was increased to 100 kHz. All data was high-pass filtered by 3 Hz. Access resistance was compensated in voltage clamp mode before switching into current clamp, where bridge balance was set to 100%. Data was not corrected for the liquid junction potential of about 15 mV.

### Recordings

Each cell was challenged with a long step current injection at various hyper and depolarizing intensities to test for the characteristic sub- and supra-threshold voltage behavior. Only neurons that generated action potentials toward depolarizing current injections were taken for further analysis. Also in each cell a −5 pA current injection between 10 and 500 ms length and 80–400 repetitions was applied. The voltage response to this hyperpolarization was used to determine membrane decay time constant (τ_*p*_) by exponential fits. Using Ohm's law the input resistance at the beginning of the hyperpolarization (*R*_*p*_) and during steady state (*R*_*s*_) was calculated. From the derived values the cell's effective capacitance *C* was calculated according to τ_*p*_ = *R*_*p*_*C*.

### ZAP stimuli

Following Puil et al. (1986), the neuronal sub-threshold resonance behavior was examined by applying a sinusoidal current *I*(*t*) with exponentially increasing frequency *f*(*t*) (ZAP-current).

$$I(t)=A\sin[\phi (t)]\;,\;f(t)={\left(2\pi \right)}^{-1}\frac{\text{d}}{\text{dt}}\phi (t)$$

The phase function is chosen such that the frequency at time 0 equals the starting frequency *f*(0) = *f*_*s*_ and after a time *D* the frequency stops at an end frequency *f*(*D*) = *f*_*e*_. In between the frequency increases exponentially in time. The function

$$\phi (t)=\frac{(2\pi )f_{s}D}{\ln(f_{e}/f_{s})}{\left(\frac{f_{e}}{f_{s}}\right)}^{t/D}$$

fulfills these three requirements. For LSO neurons in mice the frequency increased from 2.5 to 280 Hz within 96 s and in rats from 2.5 to 280 Hz, 2.5 to 300 Hz, or 2.5 to 330 Hz always within 96 s. For gerbil VNLL neurons the ZAP frequency increased from 1.8 to 260 Hz within 99 s. For P15 MSO neurons the stimulation increased from 2.5 to 330 Hz within 96 s. For MSO neurons of P60 or older animals the ZAP stimulation increased from 4 to 700 or 10 to 850 Hz over 99 s. The duration of ZAP currents was chosen long enough to approximate quasi-stationary membrane oscillations on the logarithmic frequency scale.

The depolarizing resonance frequency *f*_*r*_ was calculated from the electrophysiological recordings by measuring the time t^*^ when the maximal depolarization occurred and converted it into the frequency *f*(t^*^) of the stimulus at this given time point.

**Figure 1:**
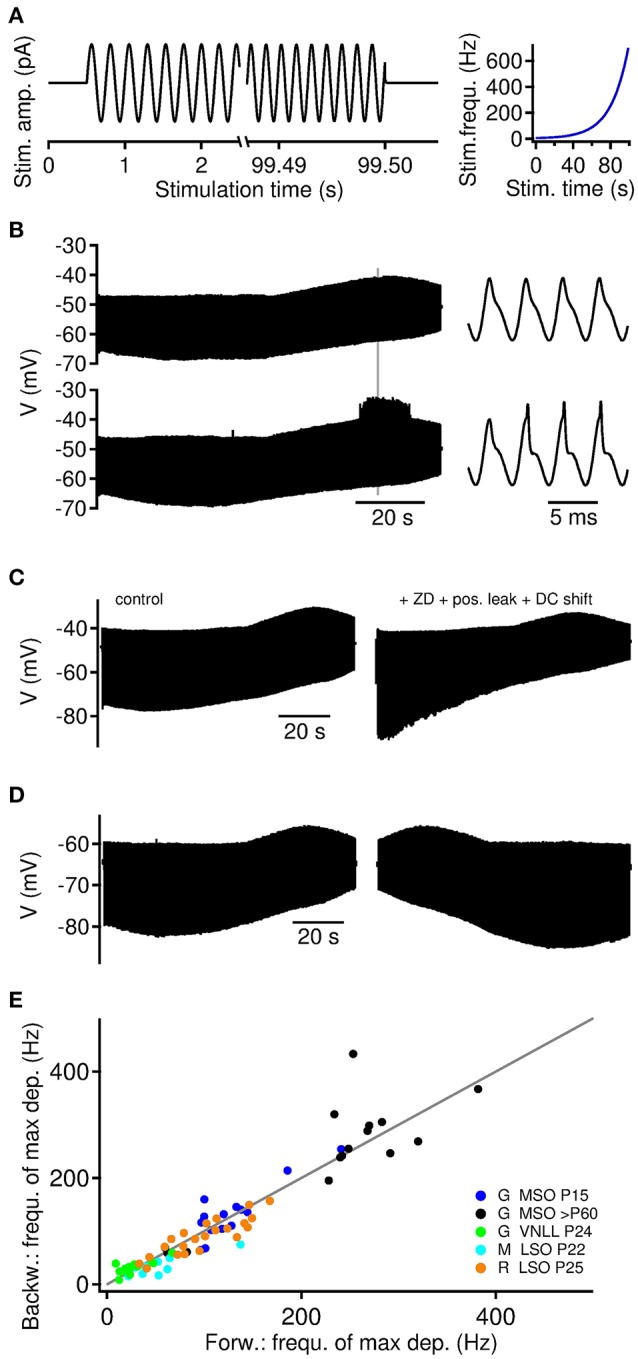
Depolarizing and hyperpolarizing membrane resonance in auditory brainstem neurons. **(A)** Current clamp stimulation paradigm. ZAP current injections of 95–99 s lengths were applied with exponentially increasing frequency between 250 and 750 Hz (right). **(B)** Sub- (top) and supra-threshold (bottom) membrane potential response to ZAP current in a >P60 MSO neuron. Hyperpolarizing and depolarizing resonances occur at different frequencies. Supra-threshold events occur at the site of depolarizing resonance. Right: Traces magnified from gray mark on the left. **(C)** Hyperpolarizing resonance depends on IH currents. In five cells the addition of ZD7288 blocked the hyperpolarizing resonance. The depolarizing resonance could be selectively recovered by holding the cell at its original resting potential (DC shift) and adding positive leak to approximate the initial resting conductance state of >P60 MSO neurons. **(D)** Membrane potential response to ZAP currents presented forward (left) and backward (right). **(E)** Depolarizing membrane resonance frequency of backward and forward presented ZAPs. Gray line indicates unity. Gerbil [G] MSO P15 *n* = 8 cells and 15 trials, MSO >P60 *n* = 7 cells and 15 trials, VNLL *n* = 10 cells and 13 trials, mouse [M] LSO *n* = 10 cells and 10 trials and rat [R] LSO *n* = 14 cells and 22 trials.

ZAP stimuli were applied for varying current amplitudes and at varying holding currents. To control for low-frequency build up of the membrane voltage during ZAP stimuli, in most cells we also applied stimuli with time-reversed frequency profile (see Figure 1D).

### Mathematical model

We assume that for small input currents *I*(*t*), the voltage follows a 2-d linear dynamics (Rotstein, 2014)

(1) $$\frac{\text{d}}{\text{d}t}\left(\begin{array}{c} C\upsilon \\ u \end{array}\right)=\left(\begin{array}{cc} -1/R_{p} & -\alpha \\ \gamma & -\beta \end{array}\right)\left(\begin{array}{c} \upsilon \\ u \end{array}\right)+\left(\begin{array}{c} I(t)\\ 0 \end{array}\right)$$

where υ denotes the voltage deflection from rest, and *u* is a relaxation variable that describes the linearized net effect of the subthreshold channels. Membrane capacitance *C* and the onset membrane resistance *R*_*p*_ comprise two physiologically interpretable parameters. Conversely, α, β, and γ are as yet unspecified parameters. All three parameters will be positive since clamping the voltage υ to a depolarized value should lead to an increase (γ > 0) in conductance up to the positive steady state value *u* = (γ/β)υ (hence β > 0). Clamping the outward conductance *u* to a positive value, however, should lead to a decrease in voltage υ = −α *R*_*p*_ *u* and thus α > 0.

In the Fourier domain, the dynamics transforms to a set of linear algebraic equations linking voltage and relaxation variable by

(iω+β)u=γυ

and, hence,

$$C\text{i}\omega v=-\upsilon /R_{p}+\frac{-\alpha \gamma }{\text{i}\omega +\beta }\upsilon +I\;.$$

Solving for υ gives υ = *Z*(ω)*I*, with the impedance

(2) $$Z(\omega )=\frac{\text{i}\omega +\beta }{(C\text{i}\omega +1/R_{p})(\text{i}\omega +\beta )+\alpha \gamma }\;.$$

The steady state input resistance *R*_*s*_ is obtained as

$$R_{s}=Z(0)=\frac{\beta }{\beta /R_{p}+\alpha \gamma }\;.$$

At the resonance frequency ω_*r*_, the modulus of the impedance is maximized,

$$\omega_{r}={\text{argmax}}_{\omega }|Z(\omega )|\;.$$

From $\frac{\text{d}}{\text{d}\omega }|Z\left(\omega \right)|=0$, we obtain this resonance frequency as

(3) $$\omega_{r}=\beta \sqrt{\left[\sqrt{{\left(\frac{1}{\beta \tau_{s}}-1\right)}^{2}-{\left(\frac{1}{\beta \tau_{p}}-1\right)}^{2}}-1\right]}\;,$$

where τ_*p*_ = *R*_*p*_*C* denotes the time constant of the onset conductance and τ_*s*_ = *R*_*s*_*C* denotes the membrane time constant resulting from the (steady state) input resistance. Both τ_*p*_ and τ_*s*_ are experimentally accessible parameters (Figure **5**) and thus the resonance frequency depends on only one free parameter β, the rate of change of the relaxation variable. Note that ω_*r*_ is the radial resonance frequency, i.e., the stimulus frequency at which the model displays the resonance equals *f*_*r*_ = ω_*r*_/(2π).

### Statistics

**Figure 2:**
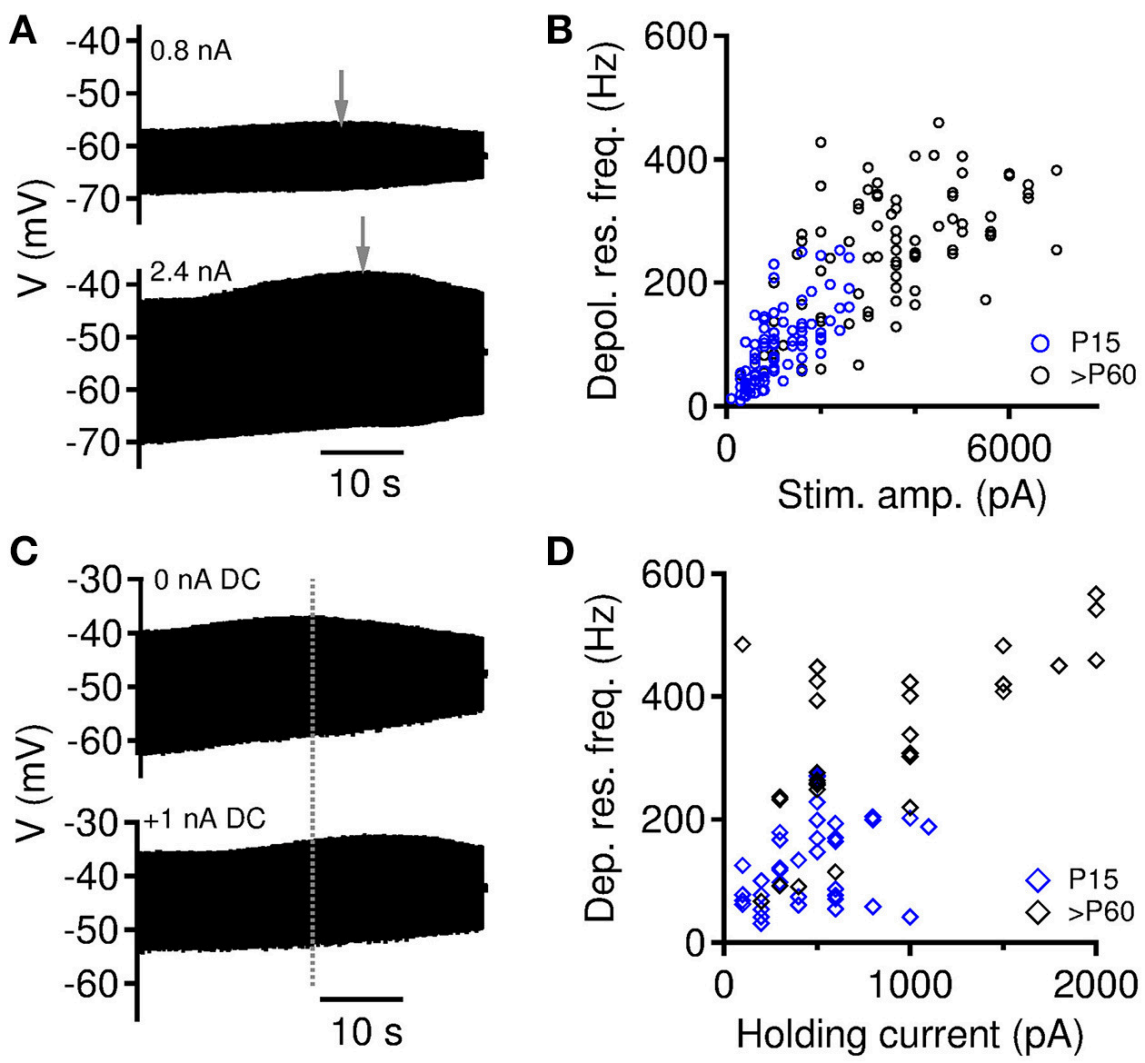
Modulation of depolarizing membrane resonance. **(A)** Depolarizing membrane resonance frequency changes with the size of injected current amplitude. **(B)** Depolarizing membrane resonance frequency increases with increasing stimulation amplitude. Data from all gerbils MSO neurons (of *n* = 14 cells all 91 trials for P15 and for *n* = 12 cells all 82 trials for >P60 are included). **(C)** Depolarizing membrane resonance frequency changes with the holding current (DC). **(D)** Depolarizing membrane resonance frequency increases with increasing DC current. Data from all gerbils MSO neurons (of *n* = 10 cells all 41 trials for P15 and for *n* = 10 cells all 32 trials for >P60 are included).

All analysis of electrophysiological data was performed in IGOR Pro (Version 6.37, Wavemetrics) and Microsoft Excel 2010. Model fitting was performed by custom-made MATLAB code. Statistical analysis of the Pearson correlation coefficient was performed by the MATLAB function corrcoef in Figure **4B** and by GraphPad Prism 7 in Figure 2.

## Results

### Resonance measurements

We probed subthreshold membrane potential resonances in auditory brainstem neurons of the medial and lateral superior olive (MSO and LSO) and the ventral nucleus of the lateral lemniscus (VNLL). To efficiently determine resonance frequencies, we applied sinusoidal current injections with exponential increasing frequency, also called ZAP stimuli (Puil et al., 1986) (Figure 1A). Using ZAP current stimuli of 96–99 second duration, neurons in all tested nuclei showed sub-threshold resonance behavior. We generally observe resonances of the hyperpolarizing troughs and the depolarizing peaks of the voltage response, where the resonance frequency of the troughs is usually lower than that of the peaks (Figure 1B). The resonance of hyperpolarizing troughs was mediated by HCN channels, which we could show using ZD7288 that selectively blocked the hyperpolarizing resonance in all five tested >P60 MSO neurons. Compensation of the holding current during ZD7288 application recovered the depolarizing membrane resonance. In one case a nearly full restoration of the depolarizing membrane resonance was achieved by adding an additional leak conductance (Figure 1C).

For larger amplitudes of the ZAP stimulus, the depolarizing resonance often facilitated the generation of action potential locked to the stimulus peaks (Figure 1B). Such a frequency dependence of supra-threshold action potential firing was observed in all neuron types. In the following we therefore focussed on the resonance frequency *f*_*r*_ of the depolarizing peaks.

To validate our experimental approach, we also applied temporally reversed ZAP inputs (Figure 1D), and found good agreement between the resonance frequencies obtained from both stimulus directions (Figure 1E).

### State-dependent resonance frequencies

In the initial data the depolarizing resonance frequency *f*_*r*_ appeared to scatter over a large range for individual neuron types (Figure 1E). In previous papers (Rotstein, 2015; Mikiel-Hunter et al., 2016), it was shown that *f*_*r*_ increases with the average membrane depolarization indicating a non-linear voltage-dependent gating of the subthreshold conductances. Thus, we expected, that our observed scatter of *f*_*r*_ can be explained by such factors as well. We therefore derived resonance frequencies for varying amplitudes *I*(*f*) of the ZAP stimulus (Figures 2A,B; 14 neurons, 91 trials of P15 MSO; 12 neurons, 82 trials of >P60 MSO) and varying magnitudes *I*_0_ of an additionally applied constant current (Figures 2C,D; 10 neurons, 41 trials of P15 MSO; 10 neurons, 32 trials >P60 MSO). As previously described (Mikiel-Hunter et al., 2016), both an increase in amplitude and an increase in *I*_0_ yielded generally larger resonance frequencies (Figures 2B,D). The correlation between resonance frequency *f*_*r*_ and amplitude was significant in all groups (Pearson's *r* = 0.71, *p* < 0.0001 for P15, *n* = 91 stimuli, *r* = 0.60, *p* < 0.0001 for P > 60, *n* = 82; *r* = 0.80, *p* < 0.0001 for both age groups, *n* = 173). The correlation between resonance frequency and holding current was only significant in *P* > 60 animals and the combined data, but not for P15 animals (Pearson's *r* = 0.06, *p* = 0.35 for P15, *n* = 41 stimuli, *r* = 0.72, *p* < 0.0001 for *P* > 60, *n* = 32; *r* = 0.60, *p* < 0.0001 for both age groups, *n* = 73), potentially indicating heterogeneous maturation states, in which some cells may still have very low densities of active channels. The holding current is thus not fully predictive for the resonance frequency.

**Figure 3:**
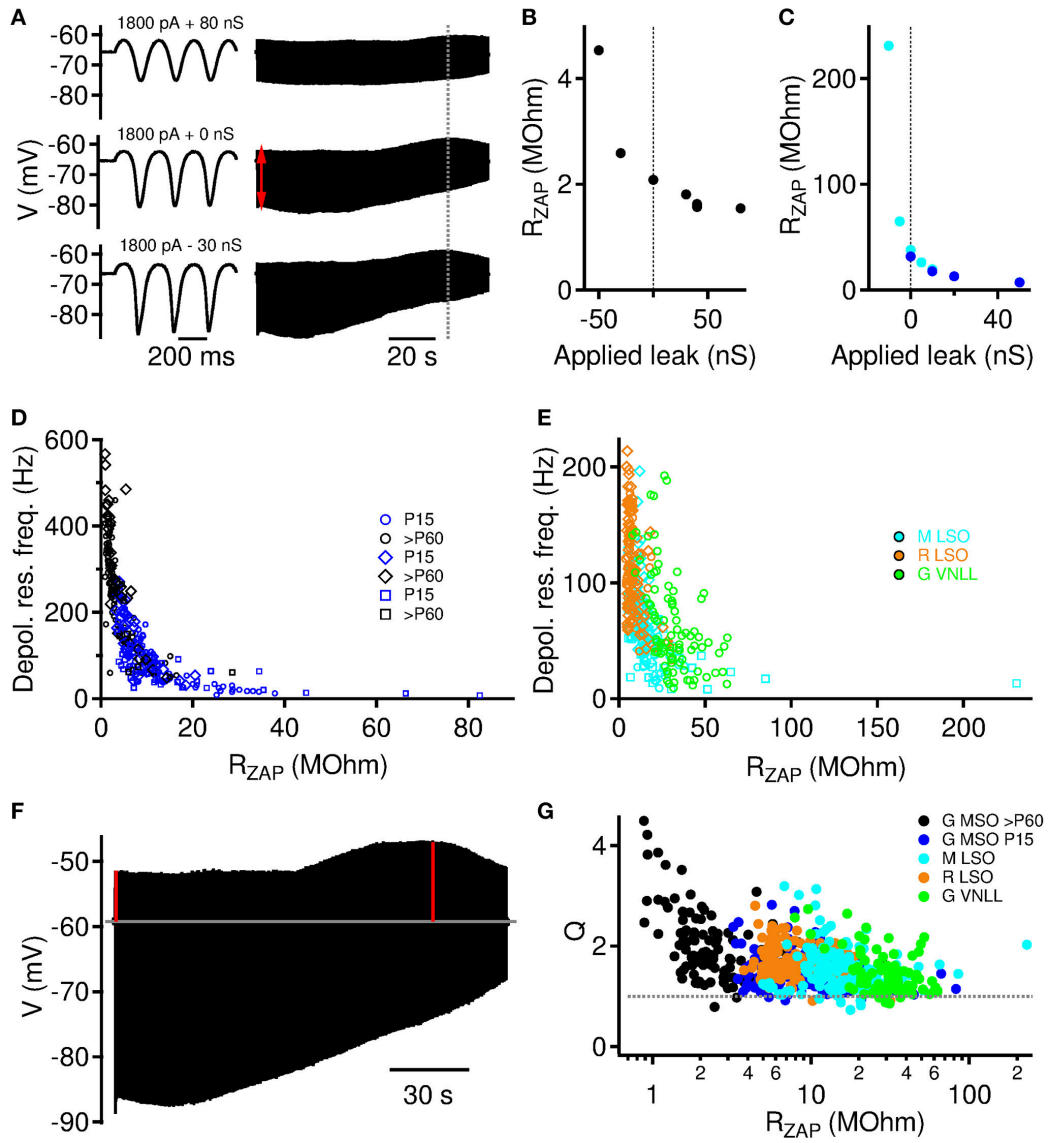
Instantaneous input resistance *R*_ZAP_ modulates depolarizing resonance. **(A)** Example traces from a >P60 MSO neuron for three different levels of artificial leak. Left: Voltage response to first three cycles. Right: whole voltage trace. Red arrow indicates position of estimated initial stimulation driven membrane resistance (*R*_ZAP_). **(B)** *R*_ZAP_ as a function of applied artificial leak for the example neuron from **(A)**. **(C)** Same as **(B)** for two further example neurons (P15 Gerbil MSO: blue, Gerbil VNLL: cyan). **(D)** Depolarizing membrane resonance frequency increases with decreasing *R*_ZAP_. Data for P15 and >P60 gerbil MSO neurons. Square symbols: trials with varying leak conductances (*n* = 6 cells, 33 trials for P15, and *n* = 3 cells, 15 trials for >P60 MSO neurons), diamonds: trials with varying holding currents, circles: trials with varying current amplitudes. **(E)** Depolarizing membrane resonance frequency increases with decreasing *R*_ZAP_. Data are from mouse (M) and rat (R) LSO and gerbil (G) VNLL. Symbols as in **(D)**. **(F)** The quality of the resonance is defined by the *Q* factor, which we compute as the ratio of the depolarizing amplitude at the resonance frequency (right vertical red line) over the depolarizing amplitude at freqeuncy 0 (left vertical red line). **(G)** *Q* factors for all recordings (varying holding current, current amplitude, and artificial leak conductance) as a function of the individual instantaneous input resistance *R*_ZAP_.

We then reasoned that if the scatter in *f*_*r*_ is due to the differential opening of subthreshold conductances, we should observe a stronger correlation by adding conductance artificially. To test this prediction we first used conductance clamp to simulate the change in input resistance and find that this manipulation indeed shifts the resonance frequency as well (Figure 3A). We assessed the effect of artificial conductance by the instantaneous input resistance $R_{\text{ZAP}}=\hat{V}/I\left(f\right)$, in which the voltage amplitude $\hat{V}$ is averaged over the first three cycles to obtain a robust estimate. When the constant background leak was increased *R*_ZAP_ decreased and the resonance frequency *f*_*r*_ increased. Conversely, when decreasing the leak, *R*_ZAP_ increased and the resonance frequency decreased (Figures 3B,C).

The definition of the instantaneous input resistance *R*_ZAP_ allowed us to compare resonance frequencies for all three stimulus paradigms shown in Figures 2, 3 and revealed a very consistent and strong correlation. We found a general monotonic decrease for MSO Neurons of both age groups (Figure 3D), exhibiting less variable resonance frequencies than in the original analyses from Figures 2B,D. Also for mouse and rat LSO and gerbil VNLL neurons (Figure 3E) we find a similiar relation, however, with VNLL data scattered more broadly along the *f*_*r*_ axes. Thus, our data indicates that the instantaneous input resistance *R*_ZAP_ is a major determinant governing the subthreshold membrane resonance frequency.

To test, whether the resonance amplitude is comparable for all probed conditions, we computed *Q* factors defined as the ratio of the depolarizing amplitude at the resonance frequency over the depolarizing amplitude at frequency 0 (Figure 3F). *Q* factors generally decreased with the instantaneous input resistance *R*_ZAP_ (Figure 3G), however, their distributions were relatively narrow and largely independent of the neuronal population except for only six *P* > 60 MSO neurons with exceptionally high values (above 3.5). For the majority of about 90% of the stimulations, the *Q* factor was between 1.05 and 2.5. The *Q* factor was above 2.5 or below 1.05 in only about 5% of the stimulations each.

### Resonance frequency decreases with input resistance

**Figure 4:**
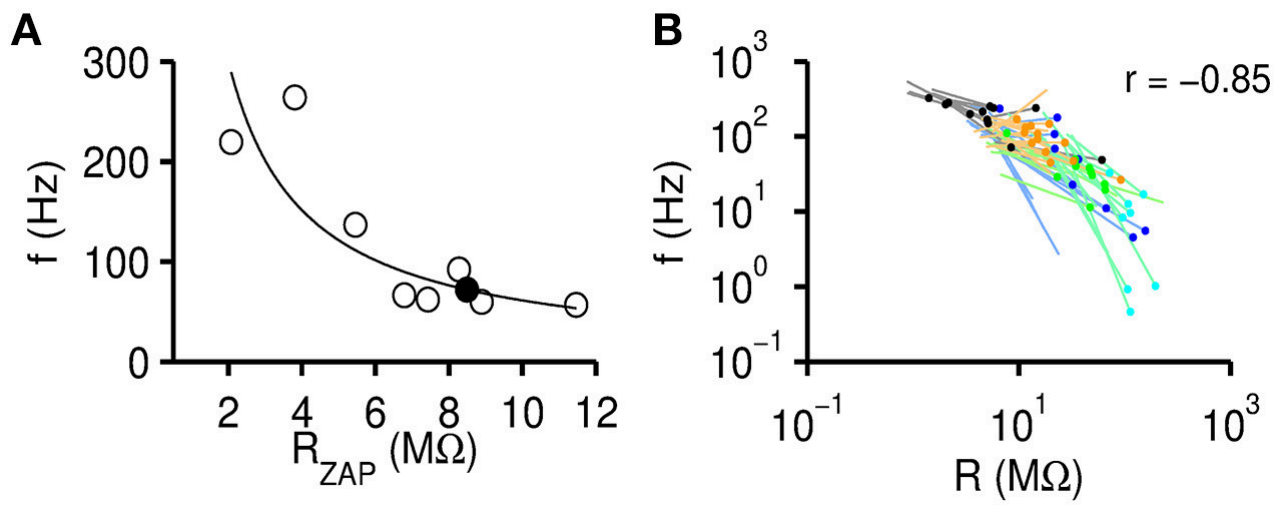
Resonance frequency negatively correlates with input resistance. **(A)** Resonance frequency *f*_*r*_ decreases with increasing instantaneous input resistance *R*_ZAP_. Black circles are data points obtained from one exemplary gerbil >P60 MSO neuron for all recording conditions [varying *I*(*f*) and *I*_0_]. The solid line fits the data by $f_{r}=a{\left(R_{\text{ZAP}}\right)}^{-b}$, with fit parameters *a* and *b*. The filled circle indicates the extrapolated resonance frequency at the steady state input resistance. **(B)** Fits (lines) and extrapolated *f*_0_ (dots) for all recorded cells (black: MSO >P60, blue: MSO P15, green: mouse LSO, cyan: gerbil VNLL, orange: rat LSO).

**Figure 5:**
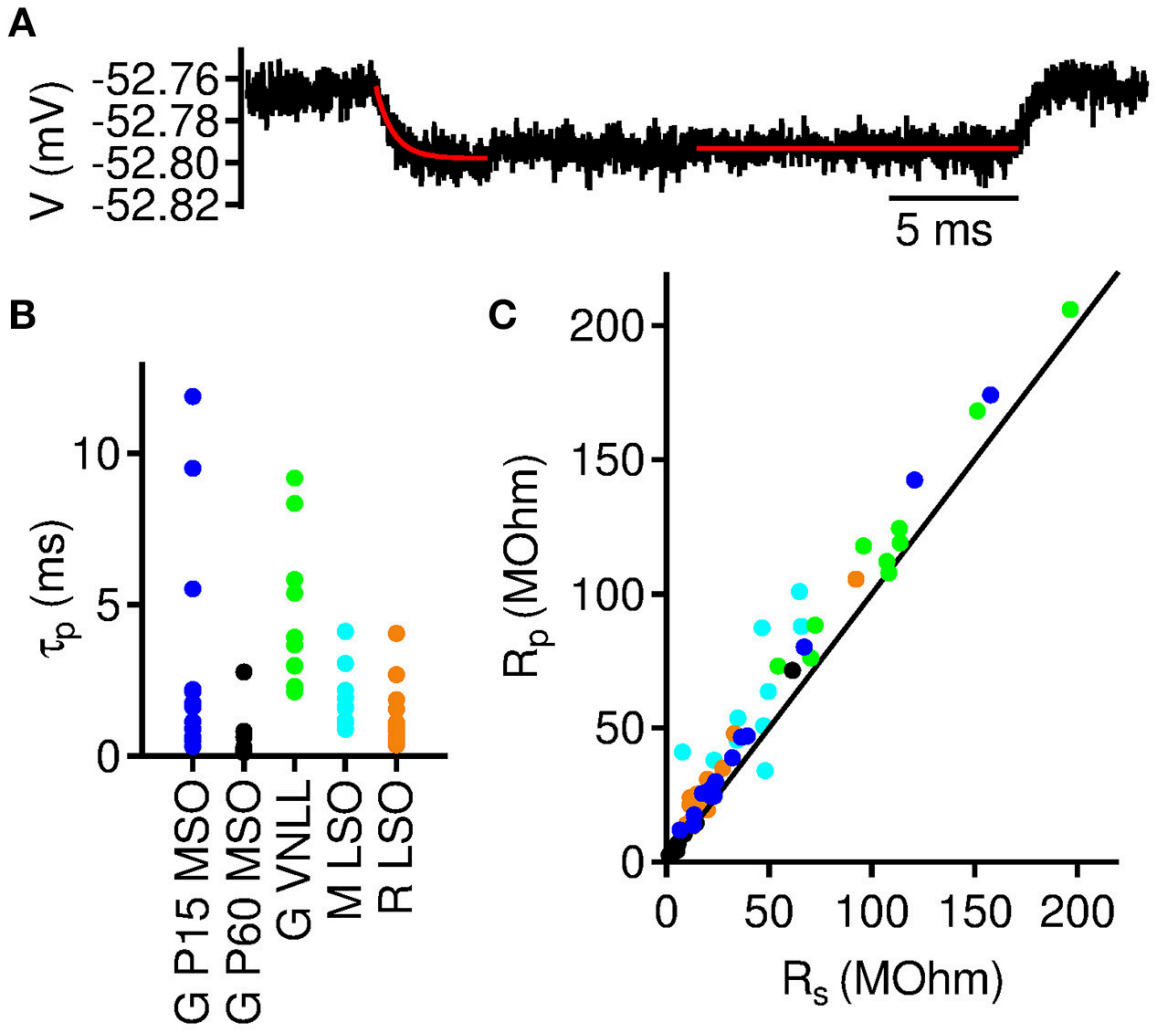
Onset and steady state membrane resistance in auditory brainstem neurons.**(A)** Averaged membrane potential response to a −5 pA current injection in a >P60 MSO neuron. Exponential fit (red line) to the initial voltage decay was used to extract membrane decay time constant (τ_*p*_) and the onset input resistance (*R*_*p*_). Straight red line indicates the region where the steady state input resistance (*R*_*s*_) was derived from. **(B)** Membrane time constants from neurons included in the analysis. **(C)** Steady state input resistance as a function of onset input resistance for all included neurons. Colors as denoted in **(B)**.

On a single cell level the relation between *R*_ZAP_ and *f*_*r*_ can be fitted by a power law $f_{r}=a{\left(R_{\text{ZAP}}\right)}^{-b}$ (Figure 4A). This dependence allowed us to identify a stimulus independent resonance frequency *f*_0_ by extrapolating *f*_*r*_ to the steady state input resistance *R*_*s*_ that is defined as the impedance value at frequency 0, *R*_*s*_ = *Z*(0). The steady state resistance *R*_*s*_ is typically close to the cell specific maximum of *R*_ZAP_ since it is defined for small current stimuli with only few additional channels opening. Our measurements thus not only allow us to define stimulus independent resonance frequency *f*_0_ but also relate it to an experimentally accessible, passive membrane property *R*_*s*_ (Figure 5A).

Resonance frequencies decrease with *R*_ZAP_ on a single cell level, but can we also find input resistance dependent effects across cells? We therefore correlated *f*_0_ with *R*_*s*_ of all cells (Figure 4B) and found a strong negative correlation (*r* = −0.85, *p* = 1.8 · 10^−15^, *n* = 52) across all experimental groups. Thus, high resonance frequencies tend to require leaky membranes, whereas low resonance frequencies are generated by non-leaky membranes. We excluded 10 out of 61 cells from this and further analyses because we considered the estimated resonance frequency *f*_0_ as too remote from the resonance frequencies *f*_*r*_ obtained for these cells from individual recordings [when min(*f*_*r*_) − *f*_0_ exceeded 33.3% of the mean *f*_*r*_].

### Theoretical model

The strong negative correlation between resonance frequency *f*_0_ and input resistance *R*_*s*_ suggests a general underlying biophysical principle. We therefore analyzed a linear dynamical model (Rotstein, 2014, 2015) to explain this general correlation (see Methods). The model consists of two coupled linear differential Equations (1) that describe the temporal evolution of small voltage deflections from rest. The model's resonance frequency from Equation (3) only depends on three biophysically defined parameters: (i) the onset membrane time constant τ_*p*_ = *CR*_*p*_, in which *R*_*p*_ denotes the peak input resistance derived from the voltage maximum upon a brief low-amplitude current stimulus (Figure 5A), (ii) the steady state membrane time constant τ_*s*_ = *CR*_*s*_, and (iii) the decay rate β of the subthreshold conductances. Whereas τ_*s*_ and τ_*p*_ are directly measurable, the last parameter β remains as a fit parameter.

### Passive membrane parameters

To fit our model, we determined τ_*p*_ (Figure 5B), and the input resistances *R*_*s*_ and *R*_*p*_ (Figure 5C) for each cell and derived its effective capacitance and steady state time constant via *C* = τ_*p*_/*R*_*p*_ and τ_*s*_ = *R*_*s*_*C*, respectively. As expected we found characteristic differences between the three different groups of cells; see Table 1.

**Table 1:** Passive parameters (mean ± s.e.m. determined from the five cell groups according to Figure 5.

	**Gerbil MSO >P60**	**Gerbil MSO P15**	**Mouse LSO**	**Rat LSO**	**Gerbil VNLL**
	**12 cells/5 animals**	**14/4**	**10/2**	**15/6**	**10/3**
*C*	41 ± 5 pF	47 ± 7 p	28 ± 4 pF	45 ± 9 pF	38 ± 5 pF
*R*_*p*_	12 ± 6 MΩ	67 ± 18 MΩ	66 ± 7 MΩ	30 ± 6 MΩ	119 ± 13 MΩ
*R*_*s*_	10 ± 6 MΩ	58 ± 16 MΩ	42 ± 6 MΩ	23 ± 6 MΩ	108 ± 14 MΩ

**Figure 6:**
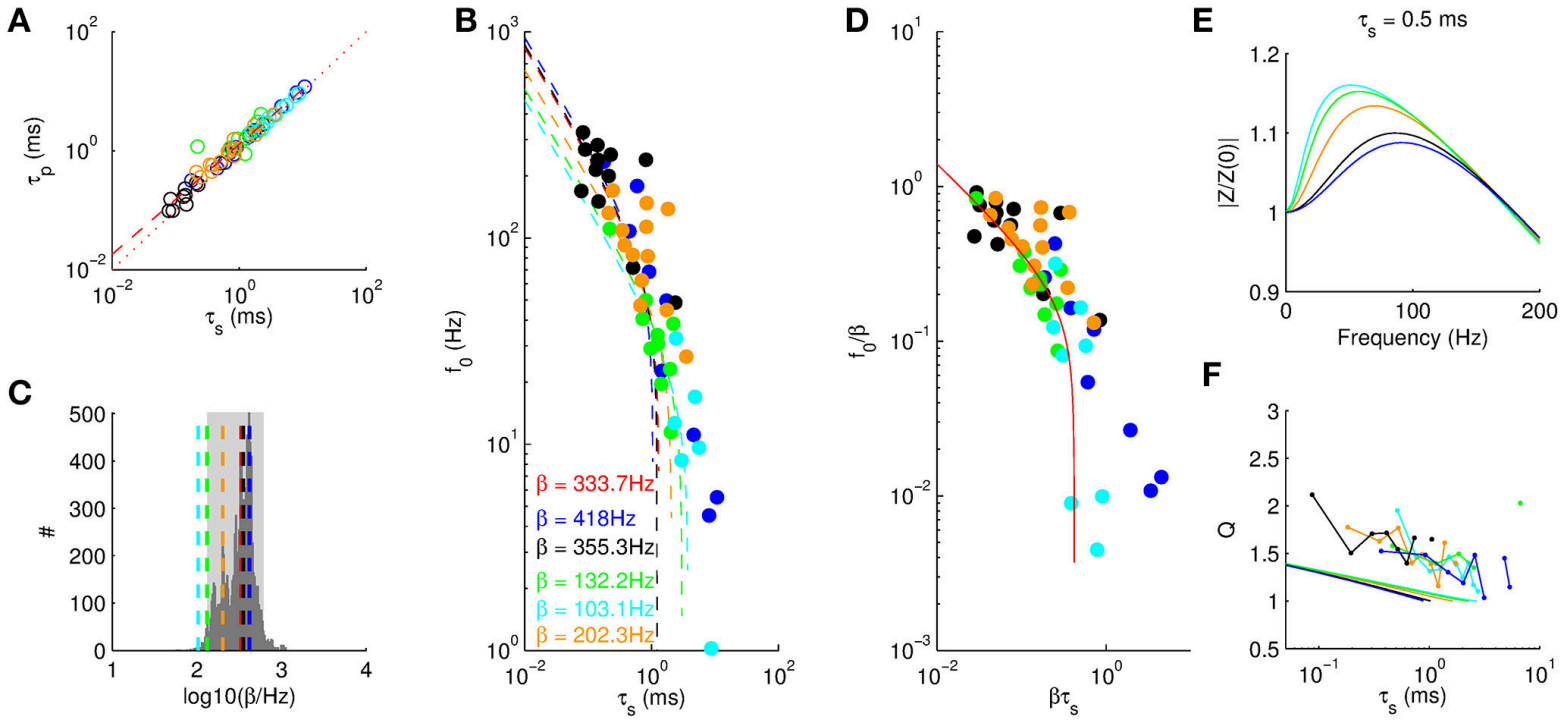
Universality of resonance behavior. **(A)** Onset time constant τ_*p*_ as a function of steady state membrane time constant τ_*s*_. The different colors identify different cell groups as in Figure 4A. Red dashed line stems from the best fit $\tau_{p}=A\tau_{s}^{B}$ (see text), dotted line depicts identity. **(B)** Resonance frequency as a function of τ_*s*_. Dashed line and β values are derived from model fits for all cell groups. Red dashed line and red β are obtained by fitting to all cell groups. **(C)** Histogram of β values obtained from fitting the model to random subsamples of all data points. Dashed lines mark β values from **(B)**. Gray area marks 95% quantile. **(D)** Rescaled resonance frequencies from Equation (3) with $\beta \tau_{p}={\left(\beta \tau_{s}\right)}^{B}\left(A\bar{\beta^{1-B}}\right)$ and $\bar{\cdot }$ denoting the average over all cell groups. **(E)** Five example amplitude profiles (τ_*s*_ = 0.5 ms) from the linear model using the cell group-specific β fitted in **(B)**. **(F)** *Q* factors from the linear model (solid lines) consistently underestimate the measured *Q* factors (dots indicate means taken in bins of 554 μs) by a factor of about 1.5.

When correlating *R*_*s*_ and *R*_*p*_ we found a strong linear correlation (Figure 5C). This is not surprising since both parameters result from the same subthreshold channels and only reflect different dynamical states. Less expectedly, the correlation seems to be largely independent of the group of cells, suggesting that the subthreshold dynamics underlying the membrane resonance are similar across all groups. We therefore generally eliminated *R*_*p*_ from the model by fitting a power law (Figure 6A)

$$\tau_{p}(\tau_{s})=0.76\cdot \tau_{s}^{0.93}\;(\text{times in seconds})\;.$$

The resonance frequency from Equation (3) thus solely depends on the steady state time constant τ_*s*_.

### Universality of membrane potential resonance

We next analyzed the dependence of resonance frequencies *f*_0_ on the steady state membrane time constant τ_*s*_. As expected *f*_0_ generally decreased with τ_*s*_. Moreover, the different cell groups showed similar dependence (Figure 6B). We thus asked, whether we could fit the model for all groups with only one parameter β. Comparing the least squares fit with the data showed indeed good agreement, for the optimal rate constant β = 333.7 Hz. We also fitted the five groups of cells independently and asked whether the variability in β would be consistent with the null hypothesis that they all arise from the same β. We therefore used 10, 000 random permutations of group labels to construct a null distribution of β (Figure 6C) and found that the mouse LSO and gerbil VNLL cells are outside the 5% significance range, whereas gerbil MSO cells and rat LSO cells could arise from the same β. We thus conclude that the different cell groups may follow the same general law, however, the mechanisms may have different molecular contributions (i.e., different heterotetrameres) and hence result in different values β for the effective channel kinetics.

To compare the quality of the fit between the different cell groups we rescaled the resonance frequencies and time constants by the group-specific fits of β (Figure 6D). We observed that only the data from juvenile (P15) MSO neurons (blue) is not completely fitted by the model. Shortly after hearing onset (P14), however, we expect a large heterogeneity in developmental states and thus a single fit parameter β might not be appropriate at this age and the scatter might simply reflect the heterogeneity in channel composition.

A further conclusion from rescaling (Figure 6D) is that we can determine the critical value τ_*c*_ for the steady state membrane time constant above which no membrane resonance is possible. This value was determined numerically as τ_*c*_ = 0.42/β. Accordingly, for neurons (like in the MSO) where the resonance is supposed to be mostly supported by Kv1 channels with kinetic constants of β^−1^ ≈ 2 ms resonances require membrane time constants below τ_*c*_ ≈ 840μs. However, if the resonance is due to channels with slower kinetics up to a range of few hundreds of milliseconds (e.g., HCN channels) the critical value increases to about τ_*c*_ ⪆ 100 ms. As a consequence, high resonance frequencies require fast channels and leaky membranes (low τ_*s*_), whereas low resonance frequencies (e.g., supporting theta oscillations) can be achieved with relatively moderate time constants and slow channels.

Finally, we computed *Q*-factors from the theoretical model (Figure 6E) and compared them with the experimentally obtained values (Figure 6F). The general decrease with τ_*s*_ was similar for both data and theory. However, the theoretical *Q*-values were only about 2/3 as large as the experimental ones indicating that our measured data also includes amplifications by non-linear channel dynamics.

## Discussion

We tested five populations of auditory brainstem neurons for membrane resonance, and found it to be strongly and robustly expressed in all of them. Since resonance frequencies depend on the level of depolarization of the membrane, we proposed to characterize them by extrapolating to the steady state input resistance, at which the resonance frequency is approximately at its minimum *f*_0_. The data fits well to a linear theory that relates *f*_0_ to *only* one easily experimentally accessible quantity, the steady state membrane time constant, and comprises a single further fit parameter, the effective relaxation rate β, which is governed by the specific molecular composition of the active subthreshold channels. The effects of β on *f*_0_ are, however, limited because of the limits to the conformational kinetics of the ion channels. Conversely, *f*_0_ changes by more than an order of magnitude as a function of the steady state membrane time constant, i.e., the leakiness of the membrane at rest.

Approximation of neural subthreshold dynamics by a 2-dimensional linear system has been previously used to efficiently describe resonance phenomena and Richardson et al. (2003) specifically indentified that leak (τ_*s*_) and coupling (β) best determine the resonance behavior. Our work shows that such linear theories can be robustly applied to data that constrain the leak variable (τ_*s*_) and thereby provide an estimate of the not directly accessible coupling variable (β).

Thus, our theory shows that a membrane potential resonance must inevitably be present in neurons where a low membrane time constant is partly due to active ion channels, whereas it cannot exist if the membrane time constants exceed a critical value of about 0.42/β (see Figure 6D). This insight has major consequence for the functional interpretation of membrane potential resonances: they virtually cannot be avoided in cells that need fast time constants, such as for example coincidence detectors. However, fine tuning of the channel kinetics (β) may allow them to be in a range at which they are most useful.

The neurons recorded in this study are binaural coincidence detectors (MSO, LSO) (Grothe et al., 2010) or involved in processing of sound envelopes (VNLL) (Zhang and Kelly, 2006; Recio-Spinoso and Joris, 2014). All of them are crucially sensitive to temporal aspects of acoustic stimuli and thus have to rely on relatively fast time constants. Sound envelope fluctuations are predominant in the range of below 10 Hertz (Fastl, 1987) and thus, unsurprisingly, VNLL neurons have resonance frequencies tuned to roughly this range and generally possess the largest time constants.

The binaural coincidence detector neurons are subdivided into MSO and LSO neurons, which encode interaural time and level differences, respectively. Gerbils are low frequency hearing rodents that in contrast to rats and mice have a large MSO and are thus used as a model system for studying interaural time-difference (ITD) encoding. The temporal acuity by which these neurons are able to distinguish ITDs is in the range of only ten microseconds (Yin and Chan, 1990; Brand et al., 2002), which necessitates the extremely fast membrane time constants of only few hundreds of microseconds in adult gerbils and causes membrane resonances in the range of 100 Hz and above. The fine structure of phase-locked inputs to MSO cells is in a range of several 100 Hz (Joris et al., 1994), and therefore these high frequency resonances boost spike rates in this frequency range (Figure 1 and Remme et al., 2014). In early postnatal stages MSO cells have slower time constants (Scott et al., 2005), which consequently leads to lower resonance frequencies. These might be crucial for allowing plasticity mechanisms to fine tune the synaptic conductances as was suggested for LSO neurons (Kotak and Sanes, 2000, 2014) rather than to enable proper ITD processing, which is predicted to be anyway impaired or even impossible owing to the slow membrane time constant.

LSO neurons in rats and mice are mostly sensitive to high frequencies (Grothe et al., 2010) at which phase-locking is reduced in the inputs (Heil and Peterson, 2015). Nevertheless LSO neurons have to be fast integrators since they have to compare ipsilateral excitation and contralateral inhibition stemming from the same envelope fluctuations. LSO neurons are indeed sensitive to binaural temporal disparities as illustrated by time intensity trading (Grothe and Park, 1995; Park et al., 1996). This need for temporal precision unavoidably endows LSO neurons with resonance properties, however, in a somewhat lower frequency range than MSO resonances. Particularly rat LSO neurons exhibit resonance frequencies of over 100 Hz. A recent study on LSO neurons (Remme et al., 2014) could not find resonances properties in high-frequency LSO cells from barely matured rats or guinea pigs. This discrepancy might partly be explained by the developmental states since for rat LSO neurons the input resistance supposedly decreases during development. However, both cell populations in Remme et al. (2014) had input resistances comparable to the range of our mouse LSO cells (but larger than our rat LSO neurons). Our model would thus predict that LSO cells from juvenile rats and guinea pigs have larger capacitance than mouse LSO neurons (and thus larger membrane time constants) such that they are no longer able to produce membrane resonance.

Mechanistically, the membrane resonances of all the auditory brainstem neurons are likely to result from HCN channels and low-threshold-activated potassium channels (Kv1). HCN-mediated subthreshold resonances in MSO neurons have first been identified in the present paper (Figure 1C). These resonances are, however, restricted to low frequencies. For MSO and LSO neurons, the presence of Kv1 channels has been extensively demnostrated (Svirskis et al., 2002; Barnes-Davies et al., 2004; Scott et al., 2005; Mathews et al., 2010). For VNLL neurons, subthreshold potassium channels have not yet fully been identified, however, Franzen et al. (2015) indicated that at least some potassium currents activate just above −50 mV. The lower β values in VNLL but also mouse LSO neurons thereby indicate that low-threshold-activated potassium conductances have a subchannel composition that is different from gerbil MSO neurons and rat LSO neurons that show faster kinetics and possibly activate at lower voltages.

Independent of the specific neuron, the present study predicts that the steady state membrane time constant (i.e., the leakiness of the neuron) is the major determinant for subthreshold membrane resonances. The leakiness of the membrane can be readily adjusted on all conceivable time scales from evolution, over development to fast activity dependent processes and thereby provides a robust, yet versatile and universal mechanism to adjust temporal processing in nerve cells.

## Author contributions

CL and FF: designed the study and wrote the paper; LF and FF: performed the experiments; CL: designed the mathematical model; All authors analyzed the data.

### Conflict of interest statement

The authors declare that the research was conducted in the absence of any commercial or financial relationships that could be construed as a potential conflict of interest.
